# Broad phenotypic impact of the effects of transgenerational heat stress in dairy cattle: a study of four consecutive generations

**DOI:** 10.1186/s12711-021-00666-7

**Published:** 2021-09-06

**Authors:** Joel Ira Weller, Ephraim Ezra, Moran Gershoni

**Affiliations:** 1 Israeli Cattle Breeders Association, 3088900 Caesarea, Israel; 2 The Volcani Center, Department of Ruminant Science, Institute of Animal Sciences, Agricultural Research Organization, 7505101 Rishon LeZion, Israel

## Abstract

**Background:**

Global warming has increased the frequency of heat stress in livestock. Although heat stress directly leads to negative effects on production and reproduction traits in dairy cattle, the transgenerational transition of these changes is poorly understood. We hypothesized that heat stress in pregnant cows might induce epigenetic modifications in the developing embryo germ cells, which, in turn, might lead to phenotypic effects in the offspring. Here, we examined whether transgenerational effects of heat stress contribute to the phenotypic expression of economic traits in Israel dairy cattle. Since heat stress in Israel occurs specifically between June and October, first we examined the association of the month of birth of F_1_ cows (pregnancy of the F_0_ dam) with the performance of the F_2_ and F_3_ female offspring. Then, we calculated an annual heat stress index and examined the association of the heat stress index during the pregnancy of the F_0_ dam with the performance of her F_2_ and F_3_ offspring. Finally, we examined intergenerational interactions of heat stress by comparing the performance of F_3_ cows according to the pregnancy seasons of the F_0_ and F_1_ animals.

**Results:**

We found a significant association of the month of birth, season of pregnancy, and heat stress index of F_0_ females, with the performance of their F_2_ and F_3_ progenies, which suggests a true transgenerational effect. The most significant transgenerational effects were on fat yield and concentration, dystocia, still-birth, and maturation.

**Conclusions:**

These findings suggest that heat stress during pregnancy affects the performance of offspring, regardless of life circumstances in at least the last three generations. Therefore, heat stress can reduce selection efficiency in breeding programs and may have economic significance in livestock.

## Background

There is growing evidence for climate change and global warming in recent decades, which include increased temperatures and a higher incidence of heat waves [1]. Environmental factors such as temperature changes can directly affect the health and productivity of dairy cows, and chronic heat stress accounts for up to 25% loss in milk production [2]. In addition, data from the Israeli herd book demonstrates a clear seasonal effect on conception rate (CR) over the two last decades, with an almost twice-higher CR during the winter compared to the summer. An average elevation of 1.5 °C during the summers of 2010, 2012, and 2015 caused an additional decrease of 5% in CR [3]. Measurements of the temperature humidity index (THI) are used to assess the risk of heat stress in livestock. There are different formulas to calculate THI, and the most common one uses the combination of temperature and relative humidity ($\mathrm{RH}$) as follows:

$$\left(1.8\times \mathrm{T}+32\right)-(0.55-0.0055\times \mathrm{RH})\times (1.8\times \mathrm{T}-26)$$

where $\mathrm{T}$ is the ambient temperature. Several studies have shown that an increase in THI modifies metabolic heat production in cattle differently depending on the cow productivity. Furthermore, breakpoints of maximal and minimal THI of 79.6 and 70.3, respectively, were shown to cause a significant acceleration in the overall death rate [4]. In addition to the direct effects of heat stress and other environmental factors on the animal, recent evidence suggests that maternal circumstances during pregnancy contribute to the phenotype of the offspring at adulthood, and may be transmitted to the next generations. For instance, a reduction in body size and in milk production during first lactation was observed for F_1_ heifers born to heat-stressed dams during late gestation, as compared to heifers born to cows of similar age and weight from calvings that were cooled during pregnancy [5, 6]. This evidence suggests that the exposure of F_1_ fetuses to heat stress during the pregnancy of their F_0_ dams can affect their phenotype in adulthood. Therefore, it is plausible that heat stress during gestation affects the epigenetics programming of the gametes in the developing fetus, and can contribute to the variation in phenotypic expression of F_2_ and F_3_ progeny.

“Transgenerational effects” are defined as observed effects on the organism that cannot be attributed to genetics or environmental effects on the individual. For example, an environmental stress can directly affect the embryo in utero and, in the female embryos, their developing oocytes. Therefore, in the case of female transmission, only altered phenotypes that occur in the third generation after exposure to environmental stress could be attributed to transgenerational inheritance. Thus if an F_0_ gestating female is exposed to environmental stress, the F_3_ generation is the first generation not directly exposed to this factor [7, 8]. Shorter timescale effects can be described as intergenerational, although they sometimes share mechanisms with transgenerational effects [9]. Evidence for intergenerational effects in cattle has recently been demonstrated in a small cohort of F_2_ granddaughters whose grandmothers were not cooled during late gestation. This study revealed a reduction in milk production and in number of days in the herd for granddaughters of not-cooled F_0_ cows, which may have caused a significant economic loss [10]. While limited in commercial populations of livestock, evidence for intergenerational effects comes mostly from studies in human and animal models. For example, Torrens et al. [11] showed that during the pregnancy of female Wistar rats, protein restriction caused elevated blood pressure and endothelial dysfunction in the F_2_ offspring throughout the maternal line. In mice, Jimenez-Chillaron et al. [12] found that maternal undernutrition during pregnancy of the F_0_ female leads to reduced birth weight, reduced glucose tolerance, and obesity in F_1_ and F_2_ offspring and that the transmission of these intergenerational effects was sex-specific and likely mediated by epigenetic modification.

Intergenerational effects in “natural populations” were demonstrated mainly in humans. Offspring of women that were pregnant during the “Dutch famine” were found to have coronary heart diseases more frequently, raised lipids, altered clotting and obesity, obstructive airways disease, microalbuminuria and decreased glucose tolerance, in a gestation-trimester dependent manner [13]. Some of these effects also affect the F_2_ offspring [14, 15]. Intergenerational effects of smoking and food supply were shown to affect F_2_ offspring in a sex-specific manner in a comparative study that was conducted within the frame of the Överkalix cohorts in northern Sweden [16].

Evidence of transgenerational effects and their impact on individual health and performance in commercial livestock populations, their effects on selection, the species evolution, and the breeding programs are sparse. In the current study, we tested the hypothesis that effects of heat stress can be trans-generationally transmitted and affect the performance for production and reproduction traits in the offspring. To this end, we analyzed hundreds of thousands of records from the Israeli herd-book over four consecutive generations, which allowed us to examine the potential transmitting ability (PTA = 1/2 of the estimated breeding value) of F_3_ cows depending on the exposure to heat stress of their F_0_ ancestors during their pregnancy.

## Methods

### Traits analyzed

We analyzed the genetic evaluation of the nine traits that are included in the Israeli breeding index, PD16, the PD16 index itself, fat and protein concentrations, and female sexual maturity. The composition of PD16 is summarized in Table 1. Fat and protein concentrations in milk, somatic cell score (SCS), female fertility and milk production persistency were evaluated as described previously [17, 18]. All the traits included in PD16, except herd-life, were analyzed by a multi-trait animal model, with each parity considered as a separate trait. In addition to the additive genetic effects, the models included the effects of herd-year-season and parity. Then, the single parity evaluations were combined into a multi-parity index as described previously [17]. Herd-life was computed as the number of days from first calving to culling, and analyzed by a single-trait animal model. For cows that were not yet culled, expected herd-life was computed as described previously [19]. First and second parity calving ease and rate of stillbirth were analyzed jointly by a multi-trait animal model including the effects of the cow calving and of the sire of calf as described by Weller and Ezra [20]. Reliabilities of all the traits analyzed by the animal model were derived as described previously [17–20]. Fat and protein concentrations were derived from the evaluations of the production traits. Female maturing rate was estimated as the number of days from birth to first insemination. Genetic evaluations were not computed for this trait, and the phenotype was analyzed.

**Table 1:** Composition of PD16, the Israeli breeding index

Trait	Index coefficient	Genetic SD	Fraction of the index^a^
Milk (kg)	0	910	0.000
Fat (kg)	8.48	32.1	0.212
Protein (kg)	21.2	22.6	0.373
Somatic cell score	− 300	0.47	0.110
Fertility (%)	26	7.15	0.145
Persistency (%)	10	5.44	0.042
Herd-life (days)	0.6	205	0.096
Maternal calving difficulty (%)	− 3	5.46	0.013
Maternal stillbirth (%)	− 6	2.21	0.010

*SD* standard deviation

^a^Computed as the product of the absolute value of index coefficient and the genetic SD for each trait, divided the sum of the index contributions for all traits

### Datasets analyzed

In order to perform the desired statistical analyses, it was necessary to generate four datasets. The numbers of records in each dataset are in Table 2. Dataset 1 (GD month) included 175,171 Israeli Holstein cows with first parity freshening dates from January 1, 2009, through December 31, 2018. Only the cows that were daughters of Israeli Holstein bulls with valid first parity records for production traits and genetic evaluations were included. In the analyses of the effects of the birth month of the maternal grand-dam (MGD) for each trait, we included only the cows for which genetic evaluations of the grand-dam were available. Similarly, in the analyses of the effects of the maternal birth month, we included only the cows for which genetic evaluations of the dam were available.

**Table 2:** Number of cows with records, dams and maternal grand-dams (MGD) included in each analysis

Data set		Trait	Dam birth month		MGD birth month	
Number	Name		Number of records	Number of dams	Number of records	Number of MGD
1	GD month	PD16	152,306	120,175	149,017	99,023
		Milk production traits^a^	175,171	134,722	171,404	109,462
		Female fertility	160,787	125,894	157,285	103,262
		Persistency	165,810	128,627	162,231	105,014
		Herd-life	174,262	134,179	170,535	109,074
		Calving traits	171,036	132,205	167,414	107,725
2	Min–Max	PD16	65,261	50,943	60,161	40,045
		Milk production traits	75,477	57,330	68,173	43,801
		Female fertility	68,996	53,400	63,176	41,572
		Persistency	71,378	54,732	65,003	42,258
		Herd-life	75,077	57,086	67,815	43,622
		Calving traits	73,662	56,255	66,730	43,182
3	Maturing	Female maturing rate	348,417	229,588	343,298	173,307
4	GD season	PD16	133,472	120,175	133,472	99,023
		Milk production traits	150,796	122,668	150,796	101,595
		Female fertility	139,698	122,668	139,698	101,595
		Persistency	144,216	122,668	144,216	101,595
		Herd-life	150,034	122,668	150,034	101,595
		Calving traits	147,644	122,668	147,644	101,595

^a^Milk, fat, and protein production; fat and protein concentration; and somatic cell score

Dataset 2 (Min–Max) included records on 75,477 cows for which the relevant ancestor, dam or grand-dam, was born from August through November. These months were selected, because preliminary results indicated that the main negative epigenetic effect was due to heat stress during the second half of the pregnancy of the relevant ancestor.

Dataset 3 (Maturing) included 348,417 virgin heifers with first inseminations between January 1, 2008 and December 31, 2017, and age at first insemination between 275 and 729 days. As in dataset 1, only the heifers that were daughters of Israeli Holstein bulls and cows were included. In the analysis of the effect of the birth month of the dams, only the heifers with valid production records for their dams were included. As in the analysis of the effect of the birth month of the MGD, only the heifers with valid production records for their grand-dams were included.

Dataset 4 (GD season) included 150,796 cows with first parity freshening dates between January 1, 2008 and December 31, 2017, with genetic evaluations for the production traits, and genetic evaluations of their dams, sires and MGD. Three birth seasons, denoted 1, 2, and 3, were defined for the dams and MGD, i.e. December through March, April through July, and August through November, respectively.

### Seasonal heat stress score

Thermal humidity index (THI) values were computed based on the ambient temperatures ($\mathrm{T}$) and the relative humidity ($\mathrm{RH}$) values recorded between 1995 and 2020 by the Israel Meteorological Service on five meteorological stations representing different climate zones in Israel: coastline and lowlands—Bet Dagan (8 km south of Tel Aviv), the inland valleys—Neve Ya’ar, the Jordan River valley—Sodom, the Arava Desert—Hazeva and the mountains—Tzuba. $\mathrm{THI}$ was calculated for each set of records as previously described [21]:

1 $$\mathrm{THI}=\left(1.8\times \mathrm{T}+32\right)-\left(0.55-0.0055\times \mathrm{RH}\right)\times \left(1.8\times \mathrm{T}-26\right).$$

To test whether one representative station could be used as an indicator of $\mathrm{THI}$ across Israel for the summer period, we computed Pearson’s correlations between equivalent $\mathrm{THI}$ measurements from the five stations. Correlations between the Bet Dagan station and the four other stations were all higher than 0.92. Based on these results, the Bet Dagan $\mathrm{THI}$ score was used as the representative $\mathrm{THI}$ for the entire country.

The seasonal heat stress score was calculated over a summer period from June 1 to September 30. Cows were assumed to be heat-stressed if the $\mathrm{THI}$ was higher than 72 [22].

### Statistical analyses

The datasets were analyzed by the general linear model procedure of the SAS software [23]. Each trait was analyzed separately. Because individual records are affected by many environmental factors, cows had different numbers of records, and because the repeat records on a cow were correlated, the dependent variable was the cow’s PTA for all traits with genetic evaluations. The model used for genetic evaluation of all the traits included the effects of herd-year-season, merit of mates and parity, except herd-life for which only a single record is generated per cow. The analysis model used for dataset 1 (GD month) to estimate the effect of the birth month of the dam was as follows:

2 $${\mathrm{PTA}}_{\mathrm{ijk}}={\mathrm{M}}_{\mathrm{i}}+{\mathrm{D}}_{\mathrm{j}}+\mathrm{DB}+\mathrm{DP}+{\mathrm{e}}_{\mathrm{ijk}},$$

where ${\mathrm{PTA}}_{\mathrm{ijk}}$ is the PTA of cow $\mathrm{k}$ for each of the traits analyzed, ${\mathrm{M}}_{\mathrm{i}}$ is the effect of birth month $\mathrm{i}$ of the cow, ${\mathrm{D}}_{\mathrm{j}}$ is the effect of birth month $\mathrm{j}$ of the cow’s dam, $\mathrm{DB}$ is the linear effect of the dam’s birth date, $\mathrm{DP}$ is the linear effect of the dam’s PTA, and ${\mathrm{e}}_{\mathrm{ijk}}$ is the random residual. Records are corrected for calving month prior to genetic evaluation, but are not corrected for birth month [17–20]. Therefore, a class effect for the cow’s birth month was included in Eq. (2). The linear effect of the dam’s birth date was included to account for genetic trend, and the effect of the dam’s PTA was included to account for possible confounding between the dam’s genetic value and her birth month. For the analysis of the MGD’s birth month, the models included the MGD’s birth month, instead of the dam’s birth month, as a class effect, and the linear effects of the MGD’s birth date and PTA. The significance of all the effects in all the models analyzed were determined by the F-test of the “Type-III” sum of squares, which is the significance of each effect relative to the residual variance after correction for all the other effects included in the model.

Four analyses were computed for dataset 2 (Min–Max) for each trait including the effect of the number of days within the birth year of the dam or grand-dam during which heat stress was recorded at the Bet Dagan weather station during the coolest time of day (MIN): THI > 72 at ~ 5:00 am and during severe heat stress (MAX): THI > 79 at any time of the day. Since these effects are only relevant for cows that are pregnant during the summer, only the records with grand-dams born in August through November were included. The numbers of days for each criterion from 1995 to 2015 are in Table 3. The analysis model for the effects of MIN and MAX on the birth year of the dam was:

**Table 3:** Number of days in the summer season in which severe heat stress (THI > 79) was measured during any time of the day (MAX), or heat stress (THI > 72) during the coolest time of day (MIN, ~ 5:00) at the Bet Dagan weather station

Year	MAX	MIN
1995	40	22
1996	46	28
1997	16	13
1998	51	34
1999	32	32
2000	41	31
2001	47	30
2002	56	42
2003	45	31
2004	42	26
2005	47	33
2006	53	28
2007	58	40
2008	55	57
2009	54	47
2010	76	74
2011	68	49
2012	87	77
2013	48	57
2014	55	52
2015	67	54

3 $${\mathrm{PTA}}_{\mathrm{ijk}}={\mathrm{M}}_{\mathrm{i}}+{\mathrm{D}}_{\mathrm{j}}+\mathrm{H}+\mathrm{B}+\mathrm{A}+{\mathrm{e}}_{\mathrm{ijk}},$$

where $\mathrm{H}$ is the linear effect of the heat stress parameter, i.e. MAX or MIN, recorded during the birth year of the grand-dam, $\mathrm{B}$ is the linear effect of the cow’s birth date, $\mathrm{A}$ is the linear effect of the dam’s age in days at the birth of her daughter, and the other terms are as defined for Eq. (2). The terms $\mathrm{B}$ and $\mathrm{A}$ were included to account for the fact that both MAX and MIN increased over the time period analyzed. The model for the effects of MIN and MAX on the birth year of the MGD was the same, except that the effect of the dam’s birth month was replaced by the effect of the MGD’s birth month, and the dam’s age at the birth of her daughter was replaced by the grand-dam’s age at the birth of her granddaughter.

In the analyses of dataset 3 (Maturing), the dependent variable was the number of days from birth to first insemination. The analysis model used for estimating the effect of the dam’s birth month was:

4 $${\mathrm{DF}}_{\mathrm{ijk}}={\mathrm{M}}_{\mathrm{i}}+{\mathrm{D}}_{\mathrm{j}}+\mathrm{B}+{\mathrm{e}}_{\mathrm{ijk}},$$

where ${\mathrm{DF}}_{\mathrm{ijk}}$ is the number of days from birth to first insemination for cow $\mathrm{k}$, and all other terms are as defined previously. To estimate the effect of the MGD’s birth month, the effect of the dam’s birth month was replaced by the effect of the MGD’s birth month. Both models included the linear effect of the heifer’s birth date to account for possible genetic trend over time.

Dataset 4 (GD season) was analyzed by two models. The first model was:

5 $${\mathrm{PTA}}_{\mathrm{ijkl}}={\mathrm{M}}_{\mathrm{i}}+{\mathrm{SD}}_{\mathrm{j}}+{\mathrm{SM}}_{\mathrm{k}}+\mathrm{B}+\mathrm{GD}+\mathrm{GS}+{\mathrm{e}}_{\mathrm{ijkl}},$$

where ${\mathrm{SD}}_{\mathrm{j}}$ and ${\mathrm{SM}}_{\mathrm{k}}$ are the class effects of the birth season of dam $\mathrm{j}$ and MGD $\mathrm{k}$, $\mathrm{GD}$ and $\mathrm{GS}$ are the PTA’s of dam $\mathrm{j}$ and sire $\mathrm{k}$ as covariates, and the other terms are as defined previously. The effects $\mathrm{B}$, $\mathrm{GD}$ and $\mathrm{GS}$ were included to account for genetic trend and possible confounding between season of birth and the genetic evaluation of the ancestors. In the second model, this dataset was analyzed including the interaction between the dam’s and MGD’s season of birth. The number of cows included in each analysis varied slightly depending on whether valid records were available for each trait for all the effects included in the analysis. The numbers of records, dams and grand-dams included in each model are in Table 2.

Frequencies of birth season of cows per birth season of their dam and grand-dam are in Table 4. The mean calving interval in Israel ranges from 410 to 430 days. For both frequencies, season 3 had the largest and season 2 the smallest number of births. This is apparently due to the significant reduction in conception rate in August and September [24], which should result in fewer births in the early summer. In both cases, the distribution of birth seasons between ancestors and progeny was not random according to the Chi-squared test (P < 0.0001), and the frequencies of ancestors and progeny being born in the same season were higher than expected by random distribution.

**Table 4:** Frequencies of birth season of cows per birth season of their dam and maternal grand-dam (MGD)

	Cow’s birth season			Total
	1	2	3	
Dam’s birth season				
1	25,249	11,104	10,810	47,163
2	6583	18,378	14,082	39,043
3	17,858	9072	38,045	64,975
MGD’s birth season				
1	18,633	12,952	15,781	47,366
2	9420	12,513	15,584	37,517
3	21,637	13,089	31,572	66,298
Total	49,690	38,554	62,937	151,181

Birth seasons: 1 from December through March; 2, from April through July; and 3, from August through November

**Fig. 1:**
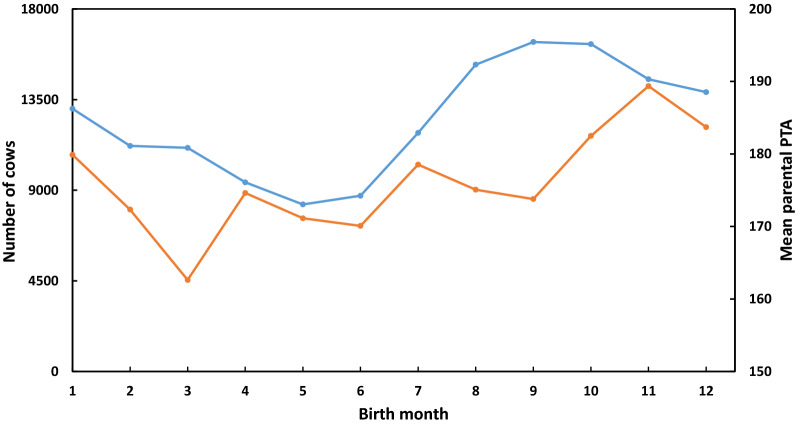
Mean potential transmitting ability (PTA) of the cows’ parents for the Israeli breeding index (PD16) and number of cows in dataset 4 per cows’ birth month. Blue line: number of cows per birth month; brown line: mean parental PTA for PD16 per birth month

Figure 1 shows the mean PTA of the cows’ parents for PD16 and the number of cows in dataset 4 that are born each month per cows’ birth month. The frequencies of births per month are in Fig. 1 with fewer births in the early summer, which as mentioned above, is due to a major reduction in conception rate in the late summer [24]. The significant differences in mean parental PTA between birth month (P < 0.0001) are somewhat surprising, and were not previously noted. It is not clear why the mean parental PTA should be highest for cows born in November and lowest for those born in March, i.e. for parents mated in February and June, respectively. Considering that 10 complete calendar years were analyzed, the general positive trend may be partly due to the mean increase of 60 PTA units in PD16 per year, which represents five units per month.

## Results

**Fig. 2:**
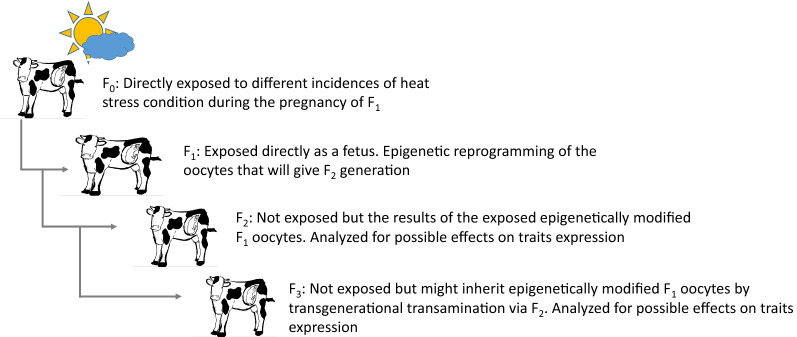
Schematic representation of the experimental model

In Israeli dairy cattle, heat stress during the day occurs mainly from June through October, as outlined in [25]. Thus, the cow’s birth month can be used as an indicator of when exposure to heat stress occurred during her dam’s pregnancy. Thus, we computed the effects of the birth month of the F_1_ and F_2_ cows (i.e. the effects of the pregnancy of the F_0_ and F_1_ cows) on the performance of F_3_ progeny (Fig. 2) after correction for confounding environment and genetic effects. Twelve production and reproduction traits and the PD16 index, were analyzed. Cow reliabilities for milk production traits ranged from 0.35 to 0.73 with a mean of 0.55, but 90% of the reliabilities were between 0.48 and 0.61. Root mean square error values and the probabilities of rejecting the null hypothesis for significance of the effects of the dams’ and maternal grand-dams’ (MGD) birth months in each analysis of datasets 1 and 3 are in Table 5. Effects of the pregnancy period of both the F_0_ and F_1_ maternal ancestors on the performance of F_3_ cows were significant for fat and protein yield, %fat, SCS, calving traits, maturing rate and the cow’s PD16 score, but not for female fertility, milk production persistency and herd-life. The effect of the birth month of the dams on milk production was significant, but not that of the MGD.

**Table 5:** Root mean square error values and probability to reject the null hypothesis for significance of the dam and maternal grand-dam (MGD) birth month effects in each analysis of data sets 1 and 3

Trait	Root mean square error^a^	Probability	
		Dam	MGD
PD16	163	10^–4^*	10^–4^*
Milk	165	10^–4^*	0.0248
Fat	8.57	10^–4^*	10^–4^*
Protein	4.58	10^–4^*	10^–4^*
Fat %	0.072	10^–4^*	0.0033*
Protein %	0.029	0.0017*	NS^b^
SCS	0.126	10^–4^*	0.0017*
Female fertility	1.58	NS	NS
Persistency	1.27	0.0341	NS
Herd-life	37.5	0.0262	NS
Dystocia	0.972	10^–4^*	10^–4^*
Stillbirth	0.645	10^–4^*	10^–4^*
Maturing rate	0.0715	10^–4^*	10^–4^*

^*^Significant after Bonferroni’s adjustment (P < 0.005); the Bonferroni correction was computed based on the 13 traits analyzed

^a^Derived from the dam analyses

^b^Not significant

**Fig. 3:**
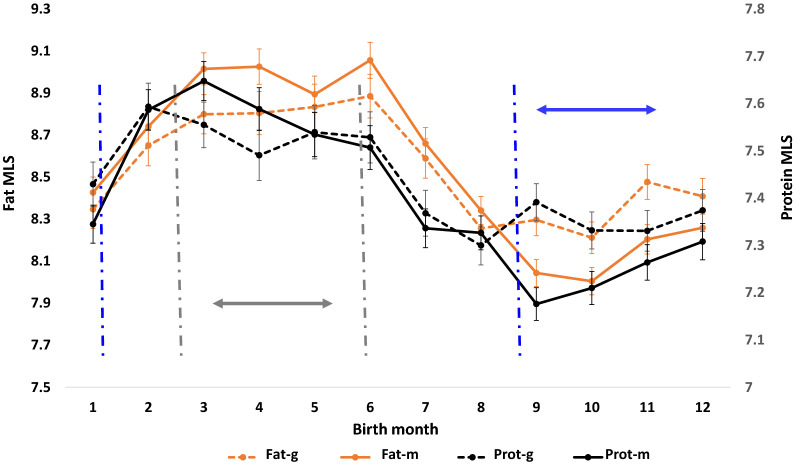
Trends in milk composition traits of the F_3_ cows per pregnancy month of their F_0_ and F_1_ ancestors. Left and right y-axes represent the mean least squares (MLS) of the potential transmitting abilities (PTA) for fat and protein yields, respectively, as functions of the birth month of the cows’ dams and maternal grand-dams. “fat-g” and “prot-g” are MLS of the cows’ fat and protein yields, respectively, as functions of the maternal grand-dams’ birth month; and “fat-m” and “prot-m” are MLS of the cows’ fat and protein yields, respectively, as functions of dams’ birth month. The error bars are the MLS standard errors. Gray and blue vertical dashed lines denote the positive (March to June) and negative (September to January) trend thresholds, respectively. Gray and blue double-headed arrows denote the boundaries of the threshold intervals

The trends of the F_3_ cows’ PTA for fat and protein yield as functions of the F_1_ and F_2_ cows' birth month are shown in Fig. 3. The PTA for fat and protein yield were higher for the F3 cows whose F_1_ and F_2_ cows were born between March to June. Thus, the corresponding pregnancy periods of the F_0_ and F_1_ cows were mainly during fall and winter, with no or little exposure to heat stress. The F_1_ and F_2_ cows that were born between August and December are the progeny of the F_0_ and F_1_ cows that had a late pregnancy during the summer and early fall, and were thus likely to have been exposed to a high incidence of heat stress conditions during the second pregnancy semester. Figure 3 shows a corresponding reduction in fat and protein yield of the F_3_ progeny of these cows. These trends are not similar to the parent average PTA for the PD16 plotted in Fig. 1.

**Fig. 4:**
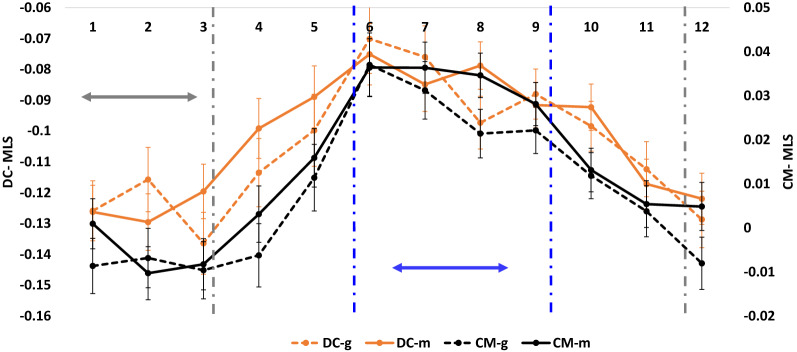
Trends in calving associated-traits of the F_3_ cows per pregnancy month of their F_0_ and F_1_ ancestors. Left and right y-axes represent the mean least squares (MLS) of potential transmitting abilities (PTA) for dystocia and stillbirth, respectively, as a function of the birth month of the cows’ dams and maternal grand-dams. “DC-g” and “CM-g” are the MLS of the cows’ dystocia and calf mortality, respectively, as functions of the maternal grand-dams’ birth month; and “DC-m” and “MC-m” are MLS of the cows’ dystocia and calf mortality, respectively, as functions of dams’ birth month. The error bars are the MLS standard errors. Gray and blue dashed lines denote positive (December to March) and negative (June to September) trend thresholds, respectively. Gray and blue arrows denote the boundaries of the threshold interval

Results for dystocia and still-birth are given in Fig. 4. Cows whose F_1_ and F_2_ ancestors were born between June and September displayed the most economically negative effect (positive values). This observation suggests a negative effect on calving traits for cows whose F_0_ and F_1_ ancestors have their last trimester pregnancy during the summer, compared to cows whose F_0_ and F_1_ ancestors have not been exposed to heat stress or have been exposed to heat stress early in gestation.

**Fig. 5:**
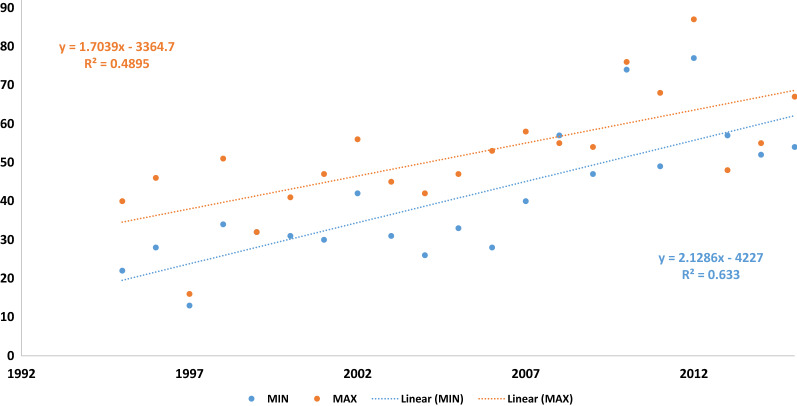
Trends in annual heat stress score per year. Dotted lines represent the maximal heat stress score (severe heat stress condition at the daily maximal temperature, orange) and the minimal heat stress score (heat stress condition at the daily minimal temperature, blue). The x-axis represents year and y-axis the computed heat stress score

To directly assess the impact of heat load during the pregnancy of the F_0_ and F_1_ cows on their progenies, we devised an annual summer heat stress score, i.e. the incidence of days with heat stress conditions per summer. Trends in annual heat stress score are plotted in Fig. 5. Analysis of the annual trends in MIN and MAX heat stress scores per year revealed a significant elevation in heat stress scores throughout the last two decades. MIN showed a greater rate of increase than MAX, with an average addition of 2 days with heat stress condition per year (r^2^ = 0.63; P = 0.0002).

The type III sum of squares F probabilities for the effect of MIN and MAX in the birth year of the dams or MGD are in Table 6. The dams’ age was significant (P < 0.05) for all traits, except for SCS. The dams’ birth month was significant (P < 0.05) for PD16, fat and protein yield, %fat, SCS and calving traits. The heat stress score of the dams’ birth year was significant for all traits, except for PD16 and herd-life, in at least one of the two analyses. The MGDs’ birth month was significant only for SCS, which is not surprising, since only the months August through November were included. The effects of the MGDs’ birth date and age were significant for all traits (P < 0.05). There was a significant association of the summer MIN scores during the F_0_ cows’ pregnancy with the performance of their F_3_ progeny for PD16, milk and fat yield, fat and protein concentration, persistency, herd-life and dystocia, but not for the other traits analyzed, including protein yield, which is the main trait under selection. Thus, annual differences in heat stress incidence during the summer experienced by the F_0_ and F_1_ cows had a general impact on performance of their progenies.

**Table 6:** Type III sum of squares F probabilities for the effect of number of days in the dam’s or maternal grand-dam’s (MGD) birth year for which heat stress (THI > 72) was recorded during the coolest time of day (MIN, ~ 5:00) or severe heat stress (THI > 79) was recorded during any time of the day (MAX) at the Bet Dagan weather station

Trait	Dam		MGD	
	MIN	MAX	MIN	MAX
PD16	NS^a^	NS	0.0060*	NS
Milk	NS	0.0001*	0.0003*	0.0329
Fat	0.0001*	0.0001*	0.0001*	0.0002*
Protein	0.0001*	0.0001*	0.0414	0.0285
Fat %	0.0001*	0.0001*	0.0001*	0.0001*
Protein %	0.0001*	NS	0.0001*	NS
SCS	0.0001*	0.0003*	0.0498	NS
Female fertility	0.0129	0.0001*	NS	0.0411*
Persistency	0.0001*	0.0001*	0.0001*	0.0002*
Herd-life	NS	0.0235	0.0001*	NS
Dystocia	0.0001*	0.0001*	0.0001*	0.0002*
Stillbirth	0.0001*	0.0001*	NS	NS

Analysis models are given in Eq. (3)

^*^Significant after Bonferroni’s adjustment (P < 0.005); the Bonferroni correction was computed based on the 13 traits analyzed

^a^Not significant

**Fig. 6:**
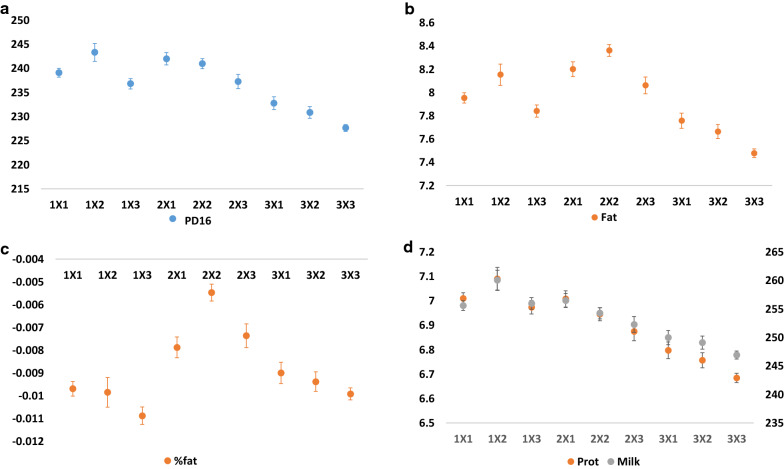
Trends in potential transmitting ability (PTA) of the F_3_ cows per combination of pregnancy seasons of F_0_ and F_1_ cows. Least square means of PTA of F_3_ cows were calculated for each combination of pregnancy seasons (x-axis, dam birth season × maternal grand-dam birth season). PD16 (**a**), protein yield (**b**), % fat (**c**) and milk and fat yield (**d**)

The models considered so far included only the main effects of the birth month of the ancestors and their exposure to heat stress conditions. However, different combinations of the pregnancy periods of the F_0_ and F_1_ cows might affect the performance of the F_3_ progenies differently, i.e., significant interactions may occur between the effects of the dam and grand-dam season of pregnancy. To answer this question, we divided the year into three birth seasons. Season 1 denoting birth during winter, with dam second-semester pregnancy during fall-winter; season 2 denoting birth season during spring with dam pregnancy mostly during fall-spring, and no overlap with summer; and season 3 denoting birth during the fall, with dam second pregnancy semester during the summer. Figure 6 shows the trends of the performance of the F_3_ cows for PD16; milk, fat and protein yield and %fat; with different combinations of pregnancy seasons of the F_0_ and F_1_ cows. The performances of the F_3_ cows according to the combinations of birth season of the F_1_ and F_2_ cows (F_0_ × F_1_ pregnancy seasons) show that the progenies’ PTA for milk, fat and protein yield; and for PD16 were affected in an additive manner. The trends in milk and protein yield are highly correlated (r^2^ = 0.98) and slightly different from those for fat yield versus milk and protein yield (r^2^ = 0.74 and r^2^ = 0.77, respectively). Positive effects on milk and protein yields were observed for combinations of birth seasons 1 and 2 of F_1_ and F_2_, where birth season of F_2_ seems dominant over that of F_1_ (i.e., exposure to heat stress during F_1_ pregnancy has a greater effect than that during F_0_ pregnancy). The most positive effect on fat yield was observed for birth season 2 of F_1_ × F_2_, which indicates mostly summer-free pregnancies for the F_0_ and F_1_ cows. However, for all these traits, we found the most negative effect on PTA of F_3_ cows for birth season 3 of F_1_ × F_2_, which indicates tandem F_0_ and F_1_ second pregnancy semesters during the summer. Nevertheless, for %fat a significant interaction between the F_0_ × F_1_ pregnancy seasons (P < 0.01) was observed, with a %fat about two-fold less negative for F_3_ whose F_1_ and F_2_ ancestors were born in season 2 (F_0_ × F_1_ summer-free pregnancies).

## Discussion

It is still an open question to what extent epigenetic mechanisms can pass on to future generations environmental effects that have been experienced by their ancestors. The Israeli dairy cattle breeding program is based on intensive data recording, including extensive pedigree, phenotypic and environmental information, which provides an opportunity to study the consequences of possible epigenetics inheritance of the cow ancestors’ environment. This allows the assessment of female transmission of transgenerational effects on the F_3_ cows originating from the exposure of the F_0_ cows to environmental stress [26]. As reflected in this analysis, the incidence of heat stress conditions in Israel has increased in the last decades (Fig. 5), likely due to global warming. Since heat stress can directly affect animal performance and lead to significant economic losses [27], this observation demonstrates the importance of studying the long-term impact of heat stress in livestock, which remains elusive. We found a significant effect of the dam’s and grand-dam’s birth month on their daughters and granddaughter’s PTA (Table 5). The average pregnancy length in cattle is ~ 280 days [28], and heat stress occurs in Israel only from June to October [25]. Thus, the cow’s birth month is an indicator of the extent of the exposure of her dam to heat stress during pregnancy. Since the statistical model is corrected for the cow’s immediate environment, the cow’s birth month, and for genetic trends, the likely explanation for the observed impact of the birth month of the F_1_ and F_2_ cows is the environment during the pregnancy of the F_0_ and F_1_ cows, specifically during the summer.

This assumption is supported by the per-month trend analysis. This analysis found that the most positive effect on production and calving traits was when the dam’s and grand-dam’s birth month was June (Figs. 3 and 4), which did not overlap with summer months during the F_0_ and F_1_ pregnancies. With respect to the most negative effect, there were slight differences between production and calving traits. The most negative effect of the dam’s and grand-dam’s birth month on production traits was during September–December (Fig. 3), which suggests a negative transgenerational impact, caused by the second half of the pregnancy of F_0_ and F_1_ cows occurring during the summer. The most negative effect of the dam’s and grand-dam’s birth month on calving traits was observed for the F_3_ cows, whose dam and grand-dams were born from December to March (Fig. 4), which suggests that exposure to heat stress during the second trimester of the pregnancy of the F_0_ and F_1_ cows negatively affects F_3_ progenies.

A recent controlled experiment of a relatively small cohort described a similar effect [10]. In this study, milk production of F_1_ and F_2_ cows born to F_0_ cows that were not cooled during late gestation was lower, providing limited evidence for an intergenerational effect of heat stress.

Although the most likely explanation for the association between the birth month of F_1_ and F_2_ cows with F_3_ performance is the effect of summer during the pregnancy of the F_0_ cows, we cannot rule out other possibilities. Thus, to provide direct evidence for the transgenerational effect of heat stress, we analyzed F_2_ and F_3_ performances according to the incidence of heat stress conditions during the pregnancy of F_0_ cows, and we found a significant association between the heat stress score during the pregnancy of the F_0_ cows and the performances of F_2_ and F_3_ progenies (Table 6). This analysis included only F_2_ and F_3_ cows, which are the descendants of F_0_ cows that underwent the second half of their pregnancy during the summer. This suggests that annual differences in heat stress incidence during the second semester pregnancy of F_0_ cows affected phenotypic expression in the F_2_ and F_3_ progenies and provided direct evidence for the broad impact of transgenerational effects of heat stress in cattle. Since the incidence of heat stress is gradually increasing, as indicated in Fig. 5, it is likely that the long-term impact of heat stress will become more prominent, and should be considered when calculating the genetic values of animals.

In the results discussed above, we analyzed only the main effects of the exposure to heat stress conditions of the F_0_ and F_1_ ancestors on their F_3_ progenies. In order for F_3_ cows to inherit the environmental effect experienced by their F_0_ ancestors, an epigenetic modification that occurred during the formation of the F_1_ gametes must be preserved, at least partially, during the formation of the F_2_ gametes [29]. Transmission of epigenetics marks could be achieved by either inefficient erasure of the gained epigenetic marks or partial restoration of a stable epigenetic domain lost because of exposure to environmental stress [29]. These possibilities raise the question of the impact of the combinations of different environmental stimuli of the F_0_ and F_1_ cows on the phenotype of the F_3_ cows. To address this question, we analyzed the performance of F_3_ cows according to different combinations of pregnancy seasons of the F_0_ and F_1_ cows. The performances of F_3_ cows for total milk, protein and fat yield per combination of pregnancy seasons of the F_0_ and F_1_ cows are additive, and that of the F_1_ seems dominant over that of the F_0_ cows (Fig. 6). This finding supports an inefficient restoration or erasure of the epigenetic marks. The tandem stimuli over two generations of heat stress are likely to maintain the epigenetics modification and thus to produce the most negative effect. The combination of different pregnancy seasons could cause partial loss or restoration of the epigenetic modification and moderate the impact.

Contrary to the additive effect observed for milk, protein, and fat yield, we found that the PTA for %fat of the F_3_ cows displayed a significant interaction with the F_0_ × F_1_ pregnancy season combination. There was a two-fold less negative effect on %fat for the F_3_ cows whose F_0_ × F_1_ have summer-free pregnancies. We suggest that this finding is not attributable to an epigenetic modification, but rather to the nature of this trait. Percentage fat is the ratio between fat and milk yields. If the correlation between the seasonal combination effects for fat and milk yield was close to unity (as observed for milk and protein yield), no transgenerational effect for %fat is expected. However, the trends for fat and milk yield are only partially correlated, and the most positive impact for fat yield is due to the season 2 × season 2 combination, which has only an intermediate effect on milk yield. Thus, increased fat yield and decreased milk yield will result in higher %fat in a pregnancy season combination-dependent manner.

## Conclusions

To the best of our knowledge, these results are the first evidence for transgenerational effects of heat stress through the maternal line on a panel of dairy cattle traits. Our results suggest that the second half of the pregnancy of F_0_ cows during the hot season causes adverse effects on production and calving traits of their F_2_ and F_3_ progeny. In addition, we found that differences in the incidence of annual heat stress conditions during pregnancies of F_0_ cows have a strong impact on the performances of F_3_ cows. We expect that this phenomenon may affect the calculation of the cows’ genetic value. The combination of different gestation periods between generations F_0_ and F_1_ is expected to moderate the impact of exposure to heat stress and can be taken into account in the reproduction strategy of the herd.

## Data Availability

The data that support the findings of this study are available from Israeli Cattle Breeders Association, but restrictions apply to the availability of these data, which were used under license for the current study, and so are not publicly available. Data are available from the authors upon reasonable request, and with permission of Israeli Cattle Breeders Association.

## References

[1] Pasqui M, Di Giuseppe E (2019). Climate change, future warming, and adaptation in Europe. Anim Front.

[2] Collier RJ, Dahl GE, Vanbaale MJ (2006). Major advances associated with environmental effects on dairy cattle. J Dairy Sci.

[3] Wolfenson D, Roth Z (2019). Impact of heat stress on cow reproduction and fertility. Anim Front.

[4] Collier RJ, Baumgard LH, Zimbelman RB, Xiao Y (2018). Heat stress: physiology of acclimation and adaptation. Anim Front.

[5] Monteiro APA, Tao S, Thompson IMT, Dahl GE (2016). In utero heat stress decreases calf survival and performance through the first lactation. J Dairy Sci.

[6] Skibiel AL, Dado-Senn B, Fabris TF, Dahl GE, Laporta J (2018). In utero exposure to thermal stress has longterm effects on mammary gland microstructure and function in dairy cattle. PLoS One.

[7] Heard E, Martienssen RA (2014). Transgenerational epigenetic inheritance: myths and mechanisms. Cell.

[8] Larson JE, Lamb GC, Funnell BJ, Bird S, Martins A, Rodgers JC (2010). Embryo production in superovulated Angus cows inseminated four times with sexed-sorted or conventional, frozen-thawed semen. Theriogenology.

[9] Perez MF, Lehner B (2019). Intergenerational and transgenerational epigenetic inheritance in animals. Nat Cell Biol.

[10] Laporta J, Ferreira FC, Ouellet V, Dado-Senn B, Almeida AK, De Vries A (2020). Late-gestation heat stress impairs daughter and granddaughter lifetime performance. J Dairy Sci.

[11] Torrens C, Poston L, Hanson MA (2008). Transmission of raised blood pressure and endothelial dysfunction to the F2generation induced by maternal protein restriction in the F0, in the absence of dietary challenge in the F1 generation. Br J Nutr.

[12] Jimenez-Chillaron JC, Isganaitis E, Charalambous M, Gesta S, Pentinat-Pelegrin T, Faucette RR (2009). Intergenerational transmission of glucose intolerance and obesity by in utero undernutrition in mice. Diabetes.

[13] Painter RC, Roseboom TJ, Bleker OP (2005). Prenatal exposure to the Dutch famine and disease in later life: an overview. Reprod Toxicol.

[14] Lumey LH (1992). Decreased birthweights in infants after maternal in utero exposure to the Dutch famine of 1944–1945. Paediatr Perinat Epidemiol.

[15] Lumey LH, Stein AD, Ravelli ACJ (1995). Timing of prenatal starvation in women and birth weight in their first and second born offspring: the Dutch famine birth cohort study. Eur J Obstet Gynecol Reprod Biol.

[16] Pembrey ME (2010). Male-line transgenerational responses in humans. Hum Fertil (Camb).

[17] Weller JI, Ezra E (2004). Genetic analysis of the Israeli Holstein dairy cattle population for production and nonproduction traits with a multitrait animal model. J Dairy Sci.

[18] Weller JI, Ezra E, Leitner G (2006). Genetic analysis of persistency in the Israeli Holstein population by the multitrait animal model. J Dairy Sci.

[19] Weller JI, Ezra E (2015). Environmental and genetic factors affecting cow survival of Israeli Holsteins. J Dairy Sci.

[20] Weller JI, Ezra E (2016). Genetic analysis of calving traits by the multi-trait individual animal model. J Dairy Sci.

[21] Honig H, Miron J, Lehrer H, Jackoby S, Zachut M, Zinou A (2012). Performance and welfare of high-yielding dairy cows subjected to 5 or 8 cooling sessions daily under hot and humid climate. J Dairy Sci.

[22] Moran J (2005). Tropical dairy farming: feeding management for small holder dairy farmers in the humid tropics.

[23] SAS Institute Inc (2013). SAS/ACCESS® 9.4 Interface to ADABAS.

[24] Weller JI, Ron M (1992). Genetic analysis of fertility traits in Israeli Holsteins by linear and threshold models. J Dairy Sci.

[25] Flamenbaum I, Galon N (2010). Management of heat stress to improve fertility in dairy cows in Israel. J Reprod Dev.

[26] Skinner MK (2008). What is an epigenetic transgenerational phenotype?. F3 or F2. Reprod Toxicol.

[27] St-Pierre NR, Cobanov B, Schnitkey G (2003). Economic losses from heat stress by US livestock industries. J Dairy Sci.

[28] Nogalski Z, Piwczyński D (2012). Association of length of pregnancy with other reproductive traits in dairy cattle. Asian-Australas J Anim Sci.

[29] Klosin A, Lehner B (2016). Mechanisms, timescales and principles of trans-generational epigenetic inheritance in animals. Curr Opin Genet Dev.

